# Cardiovascular Sequelae of the COVID-19 Vaccines

**DOI:** 10.7759/cureus.82041

**Published:** 2025-04-10

**Authors:** James N Nitz, Kylie K Ruprecht, Lukas J Henjum, Andrew Y Matta, Barnabas T Shiferaw, Zoie L Weber, Jalon M Jones, Raven May, Carmen J Baio, Kenneth J Fiala, Alaa A Abd-Elsayed

**Affiliations:** 1 Department of Anesthesiology, University of Wisconsin School of Medicine and Public Health, Madison, USA; 2 Department of Anesthesiology, Loyola University Parkinson School of Health Sciences, Madison, USA

**Keywords:** cardiovascular adverse effects, covid-19 vaccine related adverse events, cvst, myocarditis, thrombosis, vaccine-induced thrombotic thrombocytopenia (vitt)

## Abstract

Vaccines against COVID-19 present a key tool in lowering the morbidity, mortality, and transmission of the disease, but they also present a strongly controversial topic. As a result, the adverse effects of the vaccine have been under scrutiny by the public eye. A comprehensive summary of the cardiovascular (CV) adverse effects of COVID-19 vaccines is vital for clinical recognition of rare adverse events, determining the public health implications, and creating a base for future research.

In May 2023, a search was conducted in the PubMed and Cochrane databases to identify literature on CV complications resulting from the COVID-19 vaccine. All articles with relevant data and discussion regarding adverse effects of the COVID-19 vaccines were included in the review. In total, 4419 articles were screened, and 166 articles were included in the review.

The vaccine-associated CV adverse events encompassed the following conditions: myocarditis, pericarditis, acute coronary syndrome, stress cardiomyopathy, hypertension, isolated tachycardia, myocardial infarction (MI) with nonobstructive coronary arteries (MINOCA), cardiac arrest, vaccine-induced thrombotic thrombocytopenia (VITT), MI, cerebral venous thrombosis (CVT), deep vein thrombosis (DVT), pulmonary embolism (PE), and other venous thrombotic disorders. Among these, myocarditis and thrombosis, especially VITT, emerged as the most frequently cited complications in the reviewed literature. Ranges of incidences for the following were recorded among the reviewed articles: myocarditis: 2 to 17 per million, VITT: 3-10 per million, CVST: 2.6-10 per million, MI: 3-4 per million.

COVID-19 vaccines entail the potential for adverse events, although at low incidence, some of which exhibit notable severity. These adverse events exhibit demographic specificity and vaccine-specific profiles. The adverse events reviewed are uniformly acute in nature. The existing body of evidence offers limited support for the assertion that COVID-19 vaccines may elevate the baseline risk of CV events in the long term. However, the available research on effects greater than six months is scarce.

## Introduction and background

Introduction

The COVID-19 pandemic has been one of the most influential events in recent history, with far-reaching consequences that continue to unfold years after its onset. While significant progress has been made in understanding SARS-CoV-2, the medical community is still striving to fully understand the extent of its long-term effects on individuals. Vaccines for COVID-19 rapidly entered production shortly after the virus emerged, and the first dose was administered in December 2020 [1]. Companies primarily responsible for vaccine development and proliferation include Johnson and Johnson, Pfizer, Moderna, and AstraZeneca. Following their introduction, approximately 5.55 billion people in the world have received at least one dose of a COVID-19 vaccine, representing around 70% of the world population [2]. Although the vaccines presented an effective tool to combat the pandemic, they were immediately surrounded with controversy- particularly due to concerns regarding potential adverse effects. 

Of the serious adverse events associated with COVID-19 vaccines, many of them are cardiovascular (CV) in nature. While these events are rare, they highlight the need for clinicians to understand their clinical presentations and potential implications to facilitate accurate diagnosis and treatment. In addition, understanding the relationship between COVID-19 vaccines and the CV system is important in evaluating the public health implications of the vaccine.

Recognition of these adverse events is essential for clinicians and although rare the number of COVID-19 vaccines administered necessitates a representation of these events in clinical practice. Understanding and recognizing the cause of such adverse events is also necessary as it may inform future vaccination schedules. Is vital that the various adverse effects associated with the vaccines are documented, particularly as many of the symptoms of the vaccine overlap with the symptoms of COVID-19 infection itself. Many of the rare, adverse events associated with the vaccine are also complications of COVID-19 infection. As a result, the root cause of adverse events can be difficult to determine. Therefore, one pertinent aspect of a comprehensive list of all CV complications associated with COVID-19 vaccines will be the ability to compare the complications, risks, and pathophysiology of COVID-19 infection. Given the number of people, the COVID-19 pandemic and the COVID-19 vaccination campaigns have reached, it is imperative that these two factors be evaluated in the light of physicians’ observations. Additionally, as COVID-19 vaccinations have made their way into seasonal immunization regimens, clinicians must be aware of rare adverse effects.

One contentious public assertion is the suggestion that COVID-19 vaccines induce lasting alterations to physiology, elevating recipients’ inherent risk for specific conditions. A particular focus of the review is the timeline of adverse events and an evaluation of any evidence for the emergence of chronic or latent conditions secondary to COVID-19 immunization. This article will serve as a foundation for future research into how COVID-19 vaccines may play a role in population health in terms of adverse CV events.

While there is a large amount of literature devoted to the safety and efficacy of various COVID-19 vaccines, an accessible and comprehensive review of all the CV complications associated with vaccination against SARS-CoV-2 is needed. This narrative review aims to summarize the existing evidence regarding the CV complications associated with the COVID-19 vaccines in order to characterize the clinical presentation and temporal association of these adverse effects and to describe the populations that they affect. This narrative review aims to serve as a practical resource for clinicians and identify critical gaps in the current literature.

## Review

Methods

A team of authors carried out a comprehensive review of the literature regarding CV sequelae following the administration of any COVID-19 vaccine in order to compile all relevant literature in a narrative review. To achieve complete comprehensiveness a systemic and reproducible method of inclusion and review was used.

**Figure 1:**
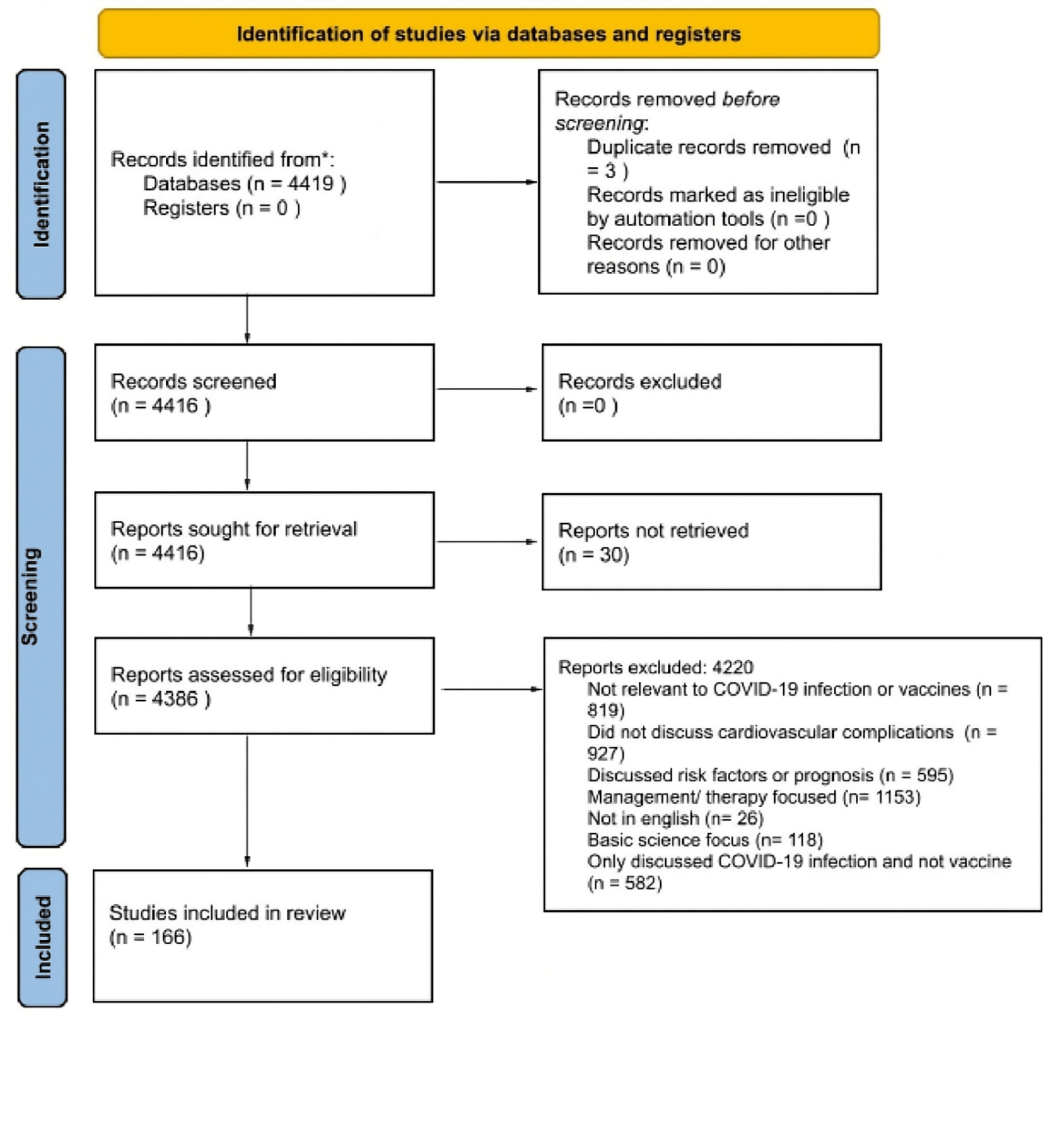
PRISMA flow diagram (2020) for cardiovascular Effects of COVID-19 vaccine review

In May 2023, a search was conducted in the PubMed and Cochrane databases to identify literature on CV complications resulting from COVID-19 infection and COVID-19 vaccines. The search employed mesh terms for all Bayesian research. The search terms used included COVID-19, Coronavirus, Covid, the novel corona "COVID-19", "Coronavirus", "Covid", "the novel corona virus", "SARS-CoV-2", ", "vaccine", "vaccination", "immunization", "inoculation", "cardiovascular complications", "stroke", "valve disease", "myocarditis", "pericarditis", "endocarditis", "arrhythmia", "hypertension", "cardiomyopathy", "thrombosis", "DVT", "PE", "VTE", "heart failure", "cardiomegaly", "cardiac disease", "heart disease", "VITT", "ITT". The process was documented in a PRISMA 2020 flowchart (Figure 1) to ensure as many relevant studies as possible were identified for the systematic review. A review of references was also conducted to retrieve additional studies following the PRISMA flow chart. Each article in the search results was evaluated for eligibility. Those that were not relevant to CV complications as a result of either COVID-19 infection or COVID-19 vaccines were excluded. All remaining articles were reviewed. Those not relating to complications due to COVID-19 infection were then removed.

The search in PubMed was as follows: ((((COVID-19[Title/Abstract] OR Coronavirus[Title/Abstract] OR Covid[Title/Abstract] OR the novel corona virus[Title/Abstract] OR SARS-CoV-2[Title/Abstract]) OR ((COVID-19[Title/Abstract] OR Coronavirus[Title/Abstract] OR Covid[Title/Abstract] OR the novel corona virus[Title/Abstract] OR AND (vaccine[Title/Abstract] OR vaccination[Title/Abstract] OR immunization[Title/Abstract] OR inoculation[Title/Abstract]))) AND (Cardiovascular Complications[Title/Abstract] OR Stroke[Title/Abstract] OR Valve disease[Title/Abstract] OR Myocarditis[Title/Abstract] OR Pericarditis[Title/Abstract] OR Endocarditis[Title/Abstract] OR Arrhythmia[Title/Abstract] OR Hypertension[Title/Abstract] OR Cardiomyopathy[Title/Abstract] OR Thrombosis[Title/Abstract] OR DVT[Title/Abstract] OR PE[Title/Abstract] OR VTE[Title/Abstract] OR Heart failure[Title/Abstract] OR Cardiomegaly[Title/Abstract] OR Cardiac Disease[Title/Abstract] OR Heart Disease[Title/Abstract] OR VITT[Title/Abstract] OR ITT[Title/Abstract])))

Articles completed between the 1st of February 2020, and the 1st of May 2023 were included. All clinical trials, meta-analyses, randomized control trials, reviews, and systematic reviews were included. Books and documents were not included in the search. The search yielded 4419 results. 

Study eligibility

Studies were eligible for inclusion if they were published in English or had an available English translation and described one or more CV adverse events occurring following the administration of any COVID-19 vaccine. The review included studies of any design, such as randomized controlled trials, cohort and case-control studies, cross-sectional studies, case series, and case reports, provided they offered clinical detail on the CV event, including symptom presentation, timing, diagnostic findings, patient demographics, or proposed pathophysiology. Articles from any country or healthcare setting were considered. Review articles were included only if they contributed unique or clinically relevant discussion of CV events post-vaccination. Additional priority was given to studies describing severe or rare adverse events, as well as those involving high-risk or special populations such as adolescents, older adults, or individuals with preexisting CV disease. Studies were excluded if they were not relevant to COVID-19 vaccination, for example, if they focused on COVID-19 infection itself or on unrelated vaccines. Studies were also excluded if they lacked a description of a CV event in a human subject, such as basic science or in-vitro research, or if they focused solely on the management or treatment of CV conditions. Articles describing events occurring in the setting of active SARS-CoV-2 infection were excluded. Articles discussing events without a clear temporal relationship to vaccination were also excluded. Additional exclusions included non-peer-reviewed material such as editorials, opinion pieces, protocols, or conference abstracts without full text; duplicate publications or secondary analyses without new findings; and articles not available in English. Relevance was assessed independently by two reviewers. In cases where both reviewers determined an article to be out of context, it was excluded. Disagreements were resolved through discussion or, if needed, adjudicated by a third reviewer.

Given the narrative nature of this review and the broad inclusion criteria, studies of various designs, including randomized controlled trials, observational studies, case series, and case reports, were included to comprehensively capture and characterize reported adverse effects of COVID-19 vaccines. As such, a formal quantitative synthesis or exclusion based on risk of bias was not conducted.

Data analysis

Each article that met the inclusion criteria was reviewed and pertinent information on the CV effects of COVID-19 and the COVID-19 vaccines were extracted and placed into a results table. Later, only articles discussing direct events from the COVID-19 vaccine were included in the results table and those discussing complications from COVID-19 infection were excluded. There was no meta-analysis of the statistics found in the articles. However, incidence, prevalence, relative risk ratios, and hazard ratios were taken from the articles when present and placed in the results table in order to describe the ranges of quantitative data reviewed.

Results

Myopericarditis

Eighty-two of the 166 articles discussed myocarditis following vaccination with mRNA-based COVID-19 vaccines [3-84]. Two articles stated that the incidence of myocarditis in healthy persons is higher than pre-pandemic numbers [33,34]. Fifty articles stated that there was a significantly higher incidence of myocarditis in younger males, ages 12-29 [3-5,7,9-15,17,18,21,23,25,27-30,32,33,36-40,42,44,45,47,49,51,54-57,62,63,67,68,70-73,75,77-79,84]. Thirty-five of the 85 articles described a higher incidence following the second dose of the vaccine [3-5,9,10,12-14,17,21,25,27,30,32,36,37,39,42,45,49,57,62,63,67,68,71-73,75,77-80,83,84]. Seven articles indicated a higher prevalence following vaccination with Pfizer (BNT162b2) compared to Moderna (mRNA-127) [4,11,36,68,71,73,83]. However, one article found more excessive events of myocarditis following the mRNA-127 vaccine than the BNT162b2 vaccine, with between 4 and 7 excess events of myocarditis and pericarditis in 28 days per 100,000 vaccinees after BNT162b2, and between 9 and 28 excess events per 100,000 vaccinees after mRNA-127 [17]. One article discussed myocarditis following adenovirus vector-based vaccines [18]. Characteristics of myopericarditis are summarized in Table 1. Ranges of incidence are summarized in Table 2.

**Table 1:** Overview of findings of cardiovascular complications of the COVID-19 vaccines from included research articles. Overview of findings of cardiovascular complications of the COVID-19 vaccines from included research articles.

Complication Subtopic	Key Findings
Myocarditis: Vaccine Association	Myocarditis following mRNA-based COVID-19 vaccines was broadly discussed in articles [3-84]. Incidence is higher in healthy males aged 12-29, especially after the second dose [3–5,7,9–15,17,18,21,23,25,27–30,32,33,36–40,42,44,45,47,49,51,54–57,62,63,67,68,70–73,75,77–79,84]. Pfizer vaccine is associated with higher prevalence in some studies [4,11,36,68,71,73,83].
Myocarditis: Incidence Rates	Incidence ranged from 2 to 17 cases per million for the general population[3,5,12,21–23,28,46,50,52,77]. Young males (16-29) reported 32-147 cases per million doses [3,5,12,21–23,28,46,50,52,77]. Many articles reported incidence rates of less than seven cases per million doses for females[3,5,12,21–23,28,46,50,52,77].
Myocarditis: Presentation	Chest pain, elevated troponin, decreased LVEF, ST elevation, and T wave abnormalities are common symptoms [7,9,11,14,18,20,23–25,44,51,54–56,60,68,72,79,84]. Myalgia, fever, and myocardial edema were reported in some cases [9,14,14,44,49,51,55,61,71,73,79]. Female patients presented with flu-like symptoms[59].
Myocarditis: Outcome	The vast majority of cases showed favorable outcomes with treatment and complete resolution of symptoms[3-84].
Myocarditis: Mechanism	Proposed mechanisms include immune-mediated responses, excess catecholamines, host miRNA release, and interactions with spike protein [14,15,20,26,31,58]. Different articles proposed various mechanisms for vaccine-induced myocarditis.
Pericarditis	Pericarditis was more highly associated with mRNA vaccines and more common in males with trends similar to myocarditis^,^ [7,8,16,19,27,41,43,48,56,64–66,70,74,81,82]. Occurs more often in older males in contrast to vaccine-related myocarditis[27,56].
Thrombosis	Thrombosis reported with both adenovirus and mRNA vaccines -vaccine-associated immune thrombosis and thrombocytopenia (VITT) was associated with adenovirus vaccines exclusively-Mechanism involves VITT[62,86,87,89–96,98–100,102–104,106–114,116–121,126,133,136–138,140,141,146–148,150,151,153,154,156,158,159,161].
Vaccine-Induced Thrombotic Thrombocytopenia (VITT): Onset	Most articles report VITT typically occurring between 5- and 42 days post-vaccination. Thrombi were often found in unusual places.
VITT: Incidence Rates	Incidence reported between 3 and 10 cases per million vaccinations [86,87]. More common in females, especially under 60 [89]. Associated with arterial and venous thrombosis[86,87,90,91,94].
VITT: Presentation	VITT is characterized by low platelet count, elevated d-dimer, and anti-PF4 antibodies[86–88,91,95,96,114] Abdominal and neurological symptoms.
VITT: Mechanism	Similar to heparin-induced thrombocytopenia (HIT), involving anti-platelet factor 4 antibodies which interact with platelets via the Fcy receptor on the surface of platelets [87,114]. Platelet activation and aggregation leading to thrombosis. A minority of patients did not show increased anti-PF4 antibodies; another proposed mechanism is the interaction of the adenovirus vector with CD46 receptors on endothelial lining[90].
Cerebral Venous Sinus Thrombosis (CVST)/Cerebral Venous Thrombosis (CVT)	CVT is reported as the primary type of acute ischemic stroke in VITT[90,91]. The incidence reported around 2.6-10 per million doses[89]. Other sites of thrombi discussed, including jugular vein, hepatic vein, and portal vein[87,94].
Other Complications	Rare complications include myocardial infarction, stress cardiomyopathy, hypertension, arrhythmias, cardiac arrest, and various thrombotic events[4,7,16,32,118,147]. Incidence and characteristics vary for each complication.

**Table 2:** Incidence rates of cardiovascular complications of the COVID-19 vaccines for groups examined and compared

Complication	Incidence Rates (Per Million Doses)	Risk Ratio, Key Findings, and Groups Compared
Myocarditis	General Population: 2 to 17 [4,7,10,14]. Young Males (16-29): 32-147 [3,5,12]	Risk ratio of 3.2 overall [3,4,5,7,10,12,14]. Risk ratio of 13.73-13.83 for males ages 16-24 [3,4,5,7,10,12,14],with higher incidence in young males[3–5,7,9–15,17,18,21,23,25,27–30,32,33,36–40,42,44,45,47,49,51,54–57,62,63,67,68,70–73,75,77–79,84]. Pfizer vaccine associated with higher risk [4,9,11]
Pericarditis	Males: Reported higher incidence[4] Older Males: Incidence comparison[27,56]	More common in males and older males[4,27,56]
Thrombosis	Adenovirus Vaccines: Varies[66,97,100,105,115,124,125,127,128,130–132,134,135,139,142,145,157,161] mRNA Vaccines: Rare[90,100,124,127]	The risk ratio for both mRNA and adenovirus vector vaccines is 1.10 [88] VITT associated with adenovirus vaccines[66,97,100,105,115,124,125,127,128,130–132,134,135,139,142,145,157,161]
VITT	3-10 [86,87]	Higher incidence in females under 60 [87,89]
CVST/CVT	2.6-10 [89,141,155]	CVT reported as primary AIS type in VITT[90,91] Incidence varies[16,19,32,86–89,91–94,96,98–100,102,106,107,111–113,116–118,125,128,129,132,136,137,141–143,146–148,150,152,154–156]
Other Complications	MI: 3-4 [4,5,8,10,16,19,32,39,62,66,147,162,163] Stress Cardiomyopathy: 0.17 [4,16,19,62,66,165] Hypertension: 13-14 [16,19,66,166]	Incidence varies for different complications

The median days from administration to onset of myocarditis reported fell between 2 and 6 days. The range of onset fell between 1 and 90 days. The duration of follow-up in the studies ranged from 21 to 183 days. The recorded duration until the resolution of myocarditis was between 6 and 7 days [37,62,73,84-133]. The ranges of onset of myopericarditis are summarized in Table 3.

**Table 3:** Ranges of time-related adverse event (ae) characteristics across all studies (Days)

Complication Class	Median AE Onset	Range of AE Onset	Study Follow-Up Period	AE Resolution
Myocarditis/Pericarditis [37,62,73,84–133]	2-6	1-90	21-183	6-7
Thrombosis (Including VITT)[16,19,55,74,86-99,101-104,109,110,112,115-123,125-129,132-135,137,139,142,143,146,148,149-152,154,155,159,161,162]	1-14	1-90	10-150	
Ischemic (MI, Stroke, CVST/CVT) [120,122,160]	1-2	1-14	21-42	

The listed incidences ranged from 2 to 35 cases per million for the general population and 32-147 cases per million doses for young males [3,5,12,21-23,28,46,50,52,77]. Three articles gave a risk ratio of 3.2 for developing myocarditis after administration of the vaccine [6,15,76]. One article gave a risk ratio of 13.73-13.83 for males ages 16-24 [17]. One article stated that the risk of myocarditis for COVID-19 infection and COVID-19 vaccination was equivalent for boys aged 12-19 [47].

Seventeen of the 85 articles on myocarditis found that the patients presented with chest pain [7,9,14,20,23,24,44,49,51,55,68,71-73,79,83,84], 17 found that patients had elevated troponin [7,11,14,18,23-25,44,51,54-56,60,68,72,79,84], 1 found that a portion of patients developed arrhythmia from myocarditis [7], 6 described patients presenting with decreased left ventricular ejection fraction (LVEF) [7,13,18,25,71,73], 15 described patients presented with ST elevation and T wave abnormalities on ECG [9,13,18,23-25,44,51,54,71-73,79,83,84], 2 found patients with myocardial edema [9,61], 3 found late gadolinium enhancement in patients [9,61,68], 6 described patients presenting with myalgia [14,44,51,55,73,79], and 5 with fever [14,44,55,71,79]. One article discussed that female patients with myocarditis from COVID-19 vaccination presented with more flu-like symptoms and less of the typical cardiac symptoms described above [59]. Eight articles discussed the fact that most vaccine-induced myocarditis cases have a favorable outcome and normalization of symptoms with treatment [34,37,38,43,45,53,60,61,72].

Many articles discussed possible mechanisms behind COVID-19 vaccine-induced myocarditis. One article discussed the histological difference between viral processes and vaccine-associated myocarditis. It stated that in this case, macrophages are the main culprit in activating the complement system instead of lymphocytes [20]. One article discussed that myocarditis may be related to excess catecholamines. It stated that the synthesized spike protein can act on adrenal chromaffin cells to increase the production of catecholamines [26]. One article proposed that the mechanism is due to the introduced mRNA causing a release of host miRNA which triggers myocarditis [58]. One article stated that because most cases occur 2-3 days after the second dose of the vaccine it is likely immune-mediated [14]. One article proposed a mechanism in which the spike proteins synthesized from the vaccine bind cardiomyocytes which are then bound by anti-spike antibodies thereby activating the classic complement system [15]. Yet another stated that excessive cytokine-mediated inflammation, autoantibody formation, and dsRNA contamination have been implicated [31].

Seventeen articles listed pericarditis as a specific complication of the mRNA COVID-19 vaccines [4,7,8,16,19,27,41,43,48,56,64-66,70,74,81,82]. Many articles reported in conjunction with myocarditis, implying that the risk factors and vulnerable populations are similar. However, two articles stated that vaccine-related pericarditis occurs largely in older males in contrast to vaccine-related myocarditis [27,56].

One article with a sample size of 5,044 looked at myocarditis after the third dose and found no cases of myocarditis or pericarditis [85].

Thrombosis

Eighty-one articles discussed thrombosis as a complication of COVID-19 vaccination [16,19,22,32,48,62,66,86-159]. Only one of the articles found that vaccines against SARS-CoV-2 were not associated with an increased risk of thromboembolism [160]. Four articles reported a general incidence of thrombosis following vaccination at around 4-280 per million [22, 113, 114,154]. One article proposed a mechanism for thrombosis following vaccination: endogenously synthesized spike proteins interact with the ACE2 receptor causing internalization and degradation which leads to platelet aggregation, thrombosis, and inflammation [101].

The reported median time until onset of thrombosis of any kind, including vaccine-induced immune thrombotic thrombocytopenia (VITT) and cerebral venous sinus thrombosis (CVST), ranged from 1 to 14 days. The shortest onset recorded was one day and the longest was 90 days. The length of follow-up in the studies ranged from 10 to 150 days [16,19,55,74,86-99,101-104,109,110,112,115-123,125-129,132-135,137,139,142,143,146,148,149-152,154,155,159,161,162].

The median duration until onset for any major ischemic event including stroke, cerebral venous thrombosis (CVT), and myocardial infarction was one to two days, with reports ranging from 1 to 14 days following vaccine administration. The length of follow-up in these studies ranged from 21 to 42 days [120,122,160].

Fifty of the 84 articles discussing VITT discussed it as a complication of specifically adenovirus vector-based vaccines [62,86,87,89-96,98-100,102-104,106-114,116-121,126,133,136-138,140,141,146-148,150,151,153,154,156,158,159,161]. An additional 19 articles discussed thrombosis with thrombocytopenia (TTS), thrombotic thrombocytopenia purpura (TTP), or immune thrombocytopenia (ITP) following primarily adenovirus vector vaccines [66,97,100,105,115,124,125,127,128,130-132,134,135,139,142,145,157,161]. Four of the articles reported specifically on thrombocytopenia following mRNA vaccination [90,100,124,127]. Many articles discussed risk factors for thrombosis following vaccination. Fifteen articles described female sex as a risk factor [86,89,92,94,99,100,107,113,128,132,135,140,143,154,161], 13 stated younger age or under the age of 60 [86,89,92,94,99,100,107,114,128,132,140,154,161], 2 stated oral contraceptives [86,87], 1 stated pregnancy [87], and 1 stated autoimmune disease [87], 1 stated estrogen supplementation [135]. Five articles discussed the incidence of VITT with estimates ranging from 1/100,000 to 1/1 million vaccine exposures [86,87,119,133,153]. One article reported that there was an increased risk of thrombosis and thrombocytopenia with viral vector vaccines compared to the Pfizer vaccine [134].

Twenty-seven of the 84 articles discussed the mechanism behind VITT. They stated that the mechanism was similar to heparin-induced thrombocytopenia (HIT). Anti-PF4 antibodies are formed causing both thrombosis and thrombocytopenia [90-93,95,96,98-100,102,103,107-109,112,117,120,131,136-139,146,147,151,158,159]. One article noted that the incidence of CVT is much higher in VITT than in HIT and proposes there may be other pathophysiological reasons for the condition [136]. One article proposed an alternative mechanism involving interactions between the adenovirus vector and CD45 receptors [103]. Another proposed mechanism is endogenously synthesized spike proteins interacting with the ACE2 receptor causing internalization and degradation which may lead to platelet aggregation, thrombosis, and inflammation [101].

Thirty-one articles listed CVST/CVT as a complication of VITT [16,19,32,86-89,91-94,96,98-100,102,106,107,111-113,116-118,125,128,129,132,136,137,141-143,146-148,150,152,154-156]. One article listed an incidence of 2.6 per million doses [89], another two listed the incidence as less than 5 per million vaccine doses [141,155]. Two articles stated that CVST/CVT was the most common complication of VITT [86,128]. Other sites of thrombi in VITT were widely discussed among the articles reviewed as well. Eight articles discussed splanchnic vein thrombosis [92,94,111-113,117,149,150], 6 articles discussed PE [62,92,113,143,147,148], 1 article discussed jugular vein thrombosis [94], 3 articles discussed portal vein thrombosis [98,144,148], 1 article discussed hepatic vein thrombosis [94], 2 articles discussed renal vein thrombosis [94,148], 1 article discussed carotid bulb thrombosis [94], 1 article discussed aortic arch and descending aorta thrombosis [94], 1 article discussed iliac vein thrombosis [94], and 1 article discussed femoral artery thrombosis [94]. Other complications of VITT were discussed as well with one article mentioning disseminated intravascular coagulation (DIC) [92], four listing hemorrhagic stroke or intracranial hemorrhage [92,94,106,146], and two articles discussing MI [118,147].

Despite the widely discussed complication of acute ischemic stroke (AIS), especially with CVT, following vaccination, one article looked at the incidence of stroke and vaccines and found that it did not appear to increase from baseline in unvaccinated individuals although none of the studies reviewed in this article looked at follow up greater than 6 months [102]. One article found that the incidence of AIS following vaccination was comparable to the general population [130]. Another article found there was an increased incidence of stroke 15-21 days following the administration of the mRNA vaccines [99].

Rare Complications

Other rarer complications of COVID-19 vaccination were discussed. Thirteen articles listed myocardial infarction or acute coronary syndrome (ACS) as a complication of COVID-19 vaccination [4,5,8,10,16,19,32,39,62,66,147,162,163]. Four articles discussed older age as a risk factor for MI following vaccination [10,39,118,162,164]. Two articles listed myocardial infarction with nonobstructive coronary arteries (MINOCA) as a complication [4,8]. Six articles listed Takotsubo cardiomyopathy as a complication of the vaccine [4,16,19,62,66,165]. Four articles listed arrhythmia as a complication [16,32,66,74], and 1 article listed tachycardia as a complication [4]. Four articles discussed new onset hypertension as a complication of the vaccine [16,19,66,166], and one gave an incidence of 3.2% [166]. Two articles described cardiac arrest as a complication of COVID-19 vaccination [32,62]. One article described the development of multisystem inflammatory syndrome in children (MIS-C) in pediatric patients receiving mRNA vaccines [45]. One article described the development of MIS in adult patients following COVID-19 vaccination [167]. One article described cardiogenic shock following COVID-19 vaccination [74]. One article described type 1 Kounis syndrome in a patient following vaccination [8]. Finally, one study found an increase in the incidence of giant cell arteritis (GCA) associated stroke in Spain following the third vaccine dose (booster) of the mRNA COVID-19 vaccine [168].

Discussion

COVID-19 vaccines are associated with rare adverse CV events. The incidence of events varies with vaccine type, vaccine brand, sex, and age of the recipient. The CV events associated with COVID-19 vaccination include: myocarditis, myopericarditis, pericarditis, ACS, Takotsubo cardiomyopathy, hypertension, isolated tachycardia, MINOCA, cardiac arrest, VITT, MI, CVST, DVT, PE and other venous thrombi.

Myocarditis

Myocarditis is a well-reported adverse event associated with mRNA COVID-19 vaccines. Across all articles common themes included: increased risk among young males, increased risk with the second dose, and increased risk with the Pfizer-Bio-N-Tech vaccine [3]. Gross estimates across all populations are around 2-5 to 17 cases per million. However, myocarditis has been shown to occur with a heavy predominance in males, especially in younger males. A total of 79-96% of all COVID-19-vaccine-induced myocarditis cases occurred in young healthy males [4,7,10,14]. The reported incidence of myocarditis varies but is reported as between 32 and 147 cases per million doses for males ages 16-29 [3,5,12]. Many articles reported the incidence among females across all ages as less than seven cases per million doses [3,5]. The risk is significantly less for older adults, with 1-2 cases per million doses for both males and females. Other trends observed are that the majority of cases occur after a second dose of the mRNA vaccine with 67-88% of cases being reported after the second dose [3,4,9]. Furthermore, there is a predominance of myocarditis following the Pfizer (BNT162b2) compared to the Moderna vaccine with around 70% of cases of myocarditis following the Pfizer vaccine [4,9,11]. The mRNA vaccines carry with them an excess risk of 1-5 cases of myocarditis per 100,000 [6].

Vaccine-induced myocarditis can present hours to weeks after the vaccine. However, the vast majority of patients develop myocarditis 2-5 days after administration [13,14]. In most studies, all patients presented with elevated troponin, and the vast majority of patients presented with chest pain. Another common sequela linked to myocarditis was decreased LVEF with reports ranging from 15% to 50% of patients with myocarditis presenting with decreased LVEF [7,13,14]. Nearly all cardiac dysfunction resolved and patients typically recovered well [9].

While myocarditis is probably the most infamous, unfortunate result of the COVID-19 vaccine, it is only the 3rd most common CV event associated with mRNA vaccination. Of the CV effects of mRNA COVID-19 vaccines, myocarditis represents about 3%, stroke about 4.3%, and thrombosis about 80% [74].

Controversy remains concerning the mechanism of myocardial damage. However, excessive cytokine-mediated inflammation, autoantibody formation due to molecular mimicry of the SARS-CoV 2 spike protein, dsRNA contamination, and immune response to mRNA have been implicated [3]. It is of note that myocarditis has been reported rarely following vaccines in the past, however, not to the extent of the mRNA COVID-19 vaccines. It is hypothesized that this is due to the increased immunogenicity and reactogenicity of this mode of vaccine.

Lastly, a common theme among the articles reviewed is a comparison between vaccine-induced myocarditis and COVID-19-infection-induced myocarditis. It is noted that the risk of myocarditis following vaccination is significantly lower than following COVID-19 infection with risk ratios of about 2-3.2 vs. 15-18.3, respectively. Although one article reports a higher level of risk among certain groups: among males from 16-24 years old the incidence rate ratio was 13.83 after the second dose of the BNT162b2 vaccines [17]. Nonetheless, COVID-19 vaccines including the mRNA vaccines remain endorsed nearly universally by the articles reviewed as a safe and effective way of preventing COVID-19 infection [3].

Pericarditis

Pericarditis is another complication associated with COVID-19 vaccination, especially mRNA vaccination. In many ways, it follows the same trends as myocarditis. Pericarditis is more common in males and more strongly associated with mRNA vaccination than adenovirus vector vaccines. Some articles state that it is more common in younger males. The median age of those who developed pericarditis was 21 with 71% of cases occurring in males [4]. Other articles state that in contrast to myocarditis following vaccination, it is more common in older males.

Thrombosis

Thrombosis is an adverse effect associated with both adenovirus vaccines and, rarely, mRNA vaccines [90]. The most common pathophysiology of thrombosis following COVID-19 vaccination is VITT. This occurs exclusively as a result of adenovirus vector-based vaccines. The risk ratio for thrombosis for both mRNA and adenovirus vector vaccines is 1: 10 [88].

Vaccine-Associated Immune Thrombosis and Thrombocytopenia (VITT)

Vaccine-associated immune thrombosis and thrombocytopenia (VITT) is one of the well-reported complications following COVID-19 vaccination. VITT has been characterized by the following laboratory abnormalities: low platelet count (< 150 × 10^9^/L, elevated d-dimer (>0.5 mg/L), and a positive test for anti-PF4 antibodies assayed with ELISA. The condition leads to thrombosis, especially venous thrombi often found in unusual places, and low platelet count which can lead to bleeding such as intracranial hemorrhage. Abdominal and/or neurological symptoms are common in presenting patients as well as symptoms such as headache, petechiae or purpura, dizziness, visual disturbances, fever, shortness of breath, hemiparesis, or pain in the back, neck, abdomen, or extremities. An autopsy study of those who died following vaccination found the main cause of death to be VITT with many patients presenting with intracranial hemorrhage and diffused microthrombi located in multiple areas.

The incidence of VITT is between 3 and 10 cases per million vaccinations [86,87]. Reports state that the Johnosn and Johnsen vaccine has a lower incidence of VITT than the AstraZeneca with a number of 2-3.2 per million and 7-10 per million, respectively. There is a female predominance, although it is not as skewed as vaccine-associated myocarditis. Almost 70-77% of cases of VITT are female. Furthermore, younger age is a risk factor with 90% of cases being reported in individuals under 60. The highest risk group of VITT and thrombosis following vaccination is females under the age of 60 on oral contraceptives [89]. Other risk factors include pregnancy and autoimmune disease [87].

The most common complication of VITT is AIS. CVT is the primary type of AIS [90,91]. Most sources report over 50% of thrombi to be CVTs while the other half occurred in other major veins [86]. Symptoms of CVST are often severe and persistent including severe headache, visual changes, altered mental status, nausea, vomiting, abdominal pain, shortness of breath, bleeding or petechial, and leg pain or swelling. CVST typically occurs in middle-aged women [93]. The incidence of CVT is reported around 2.6-10 per million doses of the vaccine administered [89]. 14% of those who developed AIS following vaccination died. Hemorrhagic stroke was also reported following vaccination both with and without CVST [89]. While ischemic stroke is usually seen in the context of VITT, it has also been reported without the presence of PF4 antibodies [92,94].

Other sites of thrombi reported include: jugular vein, hepatic vein, iliac vein, ophthalmic vein, medial gastrocnemius vein, renal vein, superficial femoral artery carotid bulb, aortic arch, descending aorta, celiac tripod, inferior mesenteric artery, portal vein, and splanchnic vein [87,94]. Bilateral adrenal hemorrhage, DIC, and ischemic bowel infarction have also been reported [87,92]. The total incidence of VTE following CHADOX1 administration was 28/100,000.

VITT has also been shown to cause arterial thrombosis. Both myocardial infarction and arterial stroke have been documented as a result of VITT [147].

The underlying mechanism is similar to HIT, as anti-platelet factor 4 (PF4) antibodies are found in patients with VITT. It is proposed that cationic PF4 proteins interact with anionic free DNA introduced in the adenovirus vaccine which creates an epitope that can be identified and bound by antibodies [87,114]. Individuals form IgG antibodies against PF4 complexes which interact with platelets via the Fcy receptor on the surface of platelets. This interaction causes increased release of PF4 leading to more complex formation, it also leads to platelet activation and aggregation leading to thrombus formation, and immune-mediated clearance of platelets leading to thrombocytopenia [86-88,91,95,96,114]. While very similar to HIT, the site on PF4 where the antibody binds differs between the two conditions. In VITT, the antibodies bind to the heparin-binding site on PF4, while in HIT, they bind to heparin-dependent antigens in other places on PF4 [88].

A minority of patients presenting with VITT-like symptoms do not show increased anti-PF4 antibodies [90]. Another proposed mechanism is the interaction of the adenovirus vector with CD46 receptors on endothelial lining that may induce thrombosis.

Rare Complications

Myocardial infarction is an uncommon adverse effect associated with COVID-19 vaccination. It has been reported following both mRNA and adenovirus vector vaccines [4,118,147]. However, there is a higher incidence following certain vaccines: more than half of the cases reported happened after vaccination with the AstraZeneca CHADOX1 vaccine [162]. In one study in the US, 86% were following the Pfizer vaccine, 11% following the J&J vaccine, and 3% following Moderna. Both typical MI and MINOCA have been reported following vaccination [4,8]. A study found that the AstraZeneca vaccine increased the risk of MI by 29% while mRNA vaccines did not carry a significant increase in risk of MI [39]. Compared to those with myocarditis, individuals who developed MI tended to be older with an average age of 63-65 and the onset following the vaccine tended to be shorter typically within 24-48 hours post-vaccination [10,39,162]. The incidence of MI has been reported as 3-4 cases per million doses administered [32]. Studies have shown a decreased risk in MI for vaccinated vs. non-vaccinated individuals during COVID-19 infection with one estimating a hazard ratio of 0.48 for the vaccinated group [39,164].

Takotsubo cardiomyopathy, or stress cardiomyopathy, is another uncommon event that can follow COVID-19 vaccine administration [4]. Studies focusing on mRNA vaccines found that the median age was around 62 years old [4]. The time from administration to first symptoms was between 15 minutes and 4 days with a mean of 2.6 days. One study found that 80% of cases following mRNA vaccination and a heavy female predominance with 90% of cases being female. It is reported that there is elevated troponin in all cases, with decreased LVEF in most cases. All cases reported recovered from their symptoms. The incidence of Takotsubo cardiomyopathy remains low with some estimates around 0.17 cases per million doses.

Hypertension has also been reported. Several case series show that COVID-19 vaccination may rarely increase blood pressure in some individuals. One case series showed that there was a >10 mmHg increase in average diastolic or systolic blood pressure in individuals during the 5 days after administration compared to before [16]. The incidence of new-onset hypertension following the administration of COVID-19 vaccines is around 13-14 cases per million doses with the majority of cases following mRNA vaccination. However, the incidence of hypertensive urgency or emergency is much lower with less than 0.5 cases per million doses [32].

A causal relationship between arrhythmia and COVID-19 vaccination has yet to be definitively evidenced. There have been reports of isolated tachycardia, sinus tachycardia, atrial fibrillation, supraventricular tachycardia, and palpitations following vaccination. Pfizer clinical trials also showed one case of paroxysmal ventricular arrhythmia [4]. Postural orthostatic tachycardia syndrome (POTS) has been noted as well, similar to a possible complication of the HPV vaccine. People propose that autoimmune action on adrenergic receptors or repression of angiotensin-II function decreases vasoconstriction and leads to tachycardia while upright [16,32]. The incidence of arrhythmias following vaccination is between 2 and 9 cases per million doses [32]. Palpitations are a well-reported event following vaccination with estimates of 28-29 cases per million doses administered [4]. Arrhythmia is also seen in cases of COVID-19 vaccine-induced myocarditis with some reports stating that 7% of these cases develop arrhythmia [7].

Cardiac arrest is another rare complication following COVID-19 vaccination. Estimates of the incidence fall between two and three cases per million doses of vaccine administered [32]. This event was primarily observed in those greater than 75 and remained very rare [62].

Public Health

The development of COVID-19 vaccines in response to the global pandemic has posed both challenges and opportunities in the fields of medicine and public health. While the COVID-19 vaccines serve as an important breakthrough in combating COVID-19 morbidity and mortality, they have also been met with a level of controversy. Among this controversy is the risk of adverse events following vaccination, prompting rigorous research to assess vaccine safety and public health implications. CV events in particular have been focused on as potential adverse events after COVID-19 vaccination. Therefore, research into a breadth of CV adverse events that occurred post-vaccination is important so that health professionals as well as patients and their families can make informed decisions regarding vaccines. In addition, having a thorough understanding of the epidemiology of post-vaccination adverse events is essential for planning and implementing public health interventions. The identification of risk factors including age, gender, vaccine type, and dose sequence, provides people with more information tailored to themselves or more specific populations. For example, recognizing that some studies have found an association between young males, particularly those aged 14-29, and a slightly increased risk of myocarditis following the second dose of mRNA vaccines could allow for early detection of symptoms in this population.

Addressing vaccine hesitancy and promoting scientifically backed vaccine information represent additional public health initiatives in the face of ongoing misinformation and skepticism surrounding COVID-19 vaccines. By being transparent about the risks and benefits of vaccination that have been found by evidence-based research, health professionals can increase trust between the general population and the healthcare sector. Putting particular emphasis on the rare rates of COVID-19 vaccine-associated CV adverse events relative to the protective impacts of the COVID-19 vaccines is vital in order to instill confidence in COVID-19 vaccines and lessen the impacts of misinformation. Additionally, the analysis of COVID-19 CV adverse effects offers insight into the cumulative risk-benefit profile of COVID-19 vaccination.

Looking at the findings of this study in a broader context, it is insightful to compare the COVID-19 vaccine-associated adverse events discussed in this study with those observed following other vaccines. For example, Carvajal et al. describe a study where between 1.5 and 2% of study participants developed CV disorders post-influenza vaccine [169]. In addition, Patja et al. conducted a study that found that the incidence of serious adverse events with possible or indeterminate causal relation with MMR vaccination was 3.2-5.3 per 100-000 vaccines [170]. Interestingly, the human papillomavirus (HPV) vaccine has also been associated with adverse events including POTS, which is CV in nature. Arana et al. report that there is approximately one POTS case reported per 6.5 million HPV vaccine doses administered in the United States [171]. These studies highlight significantly higher rates of post-vaccination adverse events compared to CV complications observed following COVID-19 vaccination in this study. It is important to note that vaccines for influenza, MMR, and HPV are widely used and generally regarded as safe by the public.

Along with comparing rates of CV adverse events after COVID-19 vaccination with other vaccine-related adverse events, it is insightful to compare them with the risks of COVID-19 infection itself. COVID-19 infection impacts the CV system, with complications including myocardial injury, myocarditis, MI, heart failure, arrhythmias, and thromboembolic events. Acute cardiac injury is the most reported CV abnormality in COVID-19, with an incidence as high as 31% of those hospitalized due to COVID-19 illness [172,173]. In addition, COVID-19 case fatality rates (CFR) have been estimated between 1.4% and 2.3% [174]. In contrast, Marchand et al. completed a pooled hazard ratio study that revealed no significant association of COVID-19 vaccination with all-cause mortality (HR = 0.89, 95% CI (0.71, 1.10), p = 0.28) [175]. While COVID-19 vaccines may have rare instances of adverse events, the overall risk is considerably lower compared to the morbidity and mortality risks associated with contracting the COVID-19 virus itself. Vaccination significantly reduces the likelihood of severe illness and death from COVID-19. These facts highlight the COVID-19 vaccines’ ability to prevent serious illness, hospitalization, and death as a result of infection, an important goal in public health globally. In conclusion, public health implications of COVID-19 vaccines include both individual risk assessment as well as population health, vaccine information and confidence, and global SARS-CoV-2 spread.

Important to note is that current research on CV adverse events following COVID-19 vaccination has been done through short-term follow-up periods, and no significant long-term follow-up period beyond around six months was found in this study. An understanding of the time-related aspects of the adverse events discussed in this paper will inform the assessment of vaccine safety and guide future research.

After reviewing all research used within this study, the median onset of myocarditis and pericarditis reported ranged from 2 to 6 days post-vaccination, with a wider span of 1 to 90 days observed across studies. The follow-up periods in these studies extended from 21 to 183 days. In most cases, the resolution of myocarditis and pericarditis cases, determined by time of onset to hospital discharge, occurred within 6 to 7 days. Within the category of thrombosis, onset was reported to occur around a median of 1 to 14 days following vaccination, with the range of onset extending up to 90 days. Studies investigating thrombosis maintained follow-up periods ranging from 10 to 150 days, a period similar to studies investigating myocarditis and pericarditis. Ischemic events such as MI and stroke also exhibit a rapid onset, with a median occurrence of 1 to 2 days post-vaccination, and a range of onset spanning from 1 to 14 days. Follow-up periods ranged from 21 to 42 days. Among all CV adverse events, the maximum length of onset of any event was 90 days with the maximum median duration until onset being 14 days. This demonstrates that of the reports available to review, all events were acute.

## Conclusions

The time-related data provided regarding CV adverse events following COVID-19 vaccination underscores the immediate concerns and management strategies surrounding these complications. However, it also reveals a gap in our understanding: the long-term implications. Despite the vast amount of information on the short-term onset and resolution of these events, there is a lack of research examining effects beyond the immediate post-vaccination period, the longest found in this study being 183 days. Without extended follow-up periods, we cannot adequately assess the persistence, recurrence, or delayed onset of complications, nor can we discern the potential contribution of COVID-19 vaccination to chronic CV conditions. This emphasizes the need for further research on the long-term implications of COVID-19 vaccination, particularly in relation to CV health. Increased long-term follow-up will ensure that patients and providers have the most accurate and up-to-date information on vaccine safety and efficacy and that public health strategies remain evidence-based.
